# Impact of Heavy Metals on the Antioxidant Activity of Vitamin D: A Metabolic Perspective

**DOI:** 10.3390/metabo15070440

**Published:** 2025-07-01

**Authors:** Ji Seo Park, Mi-Ri Gwon, Jae Hwa Lee, Jin Ju Park, Hae Won Lee, Duk-Hee Lee, Sook Jin Seong, Young-Ran Yoon

**Affiliations:** 1 Department of Molecular Medicine, School of Medicine, Kyungpook National University, Daegu 41944, Republic of Korea; jiseopark1108@gmail.com (J.S.P.); miri.gwon@gmail.com (M.-R.G.); woghk0202@gmail.com (J.H.L.); pjinju@knuh-ext.kr (J.J.P.); haewonbbc@gmail.com (H.W.L.); 2 Department of Clinical Pharmacology and Therapeutics, Kyungpook National University Hospital, Daegu 41944, Republic of Korea; 3 Clinical Omics Institute, School of Medicine, Kyungpook National University, Daegu 41405, Republic of Korea; 4 Department of Preventive Medicine, School of Medicine, Kyungpook National University, Daegu 41944, Republic of Korea; lee_dh@knu.ac.kr

**Keywords:** vitamin D deficiency, heavy metals, reactive oxygen species, docosahexaenoic acid

## Abstract

**Background/Objectives**: Vitamin D (VD) is metabolized in the body and plays a crucial role in regulating the antioxidant system. While exposure to heavy metals (HMs) inhibits VD activity, HMs can also be absorbed following VD stimulation. Despite differing views on the interaction between HM and VD activity, the effects of HM exposure on VD-related pathways have not been examined using metabolomics. This study aimed to investigate the impact of HM exposure on VD-related antioxidant activity under VD deficiency conditions using untargeted metabolic profiling. **Methods**: In this retrospective cohort study, 46 plasma samples were analyzed using ultra-high-performance liquid chromatography coupled with quadrupole time-of-flight mass spectrometry (UHPLC-QTOF/MS). Metabolic profiling was performed on two groups: individuals with severe VD deficiency and low HM exposure (SVDD–LHM) and those with VD deficiency and high HM exposure (VDD–HHM). **Results**: As a compensatory response to oxidative stress induced by HMs, VD-related antioxidant pathways may be associated with elevated levels of antioxidants, including bilirubin, eicosapentaenoic acid (EPA), and docosahexaenoic acid (DHA). In-creases in EPA and DHA were also linked to alterations in lipid metabolism, including diacylglycerol and phosphatidylcholine levels. DHA showed an area under the curve (AUC) of 0.850 (95% CI: 0.651–0.990), suggesting that DHA could serve as a potential biomarker for VD activity in response to HM exposure. **Conclusions**: The identified metabolites and metabolic pathways suggest that HM exposure may stimulate VD-related antioxidant activity, even under VD-deficient conditions.

## 1. Introduction

Vitamin D (VD) regulates energy homeostasis as well as endocrine and antioxidant systems [1,2]. Consequently, VD deficiency (VDD) is a global health concern because it elevates the risk of musculoskeletal and cardiometabolic diseases [2]. Although the definition of VDD remains a topic of debate among expert groups, serum VD concentrations above 30 ng/mL are generally considered sufficient [3]. VDD is commonly defined as a serum 25(OH)D level below 30 ng/mL, and severe vitamin D deficiency (SVDD) refers to levels below 10 ng/mL [4,5,6].

Heavy metals (HMs) can enter the body through contaminated food and water, polluted air, or dermal exposure. HMs bioaccumulate by forming covalent bonds with organic molecules, and their toxic effects intensify with increased exposure [7]. The most hazardous HMs to human health include arsenic (As), cadmium (Cd), chromium (Cr), lead (Pb), and mercury (Hg) [8]. Among these, Cd is notably toxic even at low concentrations [9]. HMs exert toxicity by generating reactive oxygen species (ROS), which cause oxidative stress and can lead to cell-membrane damage, nucleic acid-DNA damage, impaired protein function, and redox imbalance [10,11].

There is conflicting evidence regarding the interaction between VD and HMs. Some studies report that HM exposure may reduce VD levels; for example, Cd exposure has been linked to lower VD levels in children [12], and inverse associations have also been observed in pregnant women [13]. However, other studies involving children and animal models have shown a tendency for HM concentrations to increase with rising VD levels [14,15]. This phenomenon may be explained by VD-induced calcium transport channels, which are also involved in HM uptake [15,16]. Based on these findings, this study hypothesizes that HMs may accumulate in a VD-dependent manner and that VD may activate antioxidant pathways to mitigate the toxic effects of ROS produced by HM exposure.

The activation of antioxidant-related metabolic pathways can be inferred from alterations in metabolite profiles, highlighting the need for a metabolomics-based approach to investigate complex physiological responses. In particular, non-targeted metabolomics enables the comprehensive metabolic profiling of both endogenous and exogenous metabolites, making it a suitable analytical strategy to explore metabolic changes associated with VD levels and HM exposure [17,18]. Although the antioxidant role of VD has been widely discussed in the literature, its interaction with HM exposure remains unclear.

Therefore, this study aimed to investigate VD-associated antioxidant pathways in relation to HM exposure using non-targeted metabolomics. Additionally, we sought to compare individuals with low VD and low HM levels to those with higher levels of both VD and HM.

This study aimed to provide metabolomics-based evidence supporting the association between increased HM levels and VD, as well as the antioxidant responses induced by VD. These results may improve understanding of the underlying mechanisms behind the antioxidant effects of VD and may identify potential biomarkers of a metabolic defense response against HM toxicity.

## 2. Materials and Methods

### 2.1. Sample Collection and Measurement

This retrospective cohort study was approved by the Institutional Review Board of KNUH (2013-12-016-002) in Daegu, Republic of Korea and was registered with the Clinical Research Information Service (https://cris.nih.go.kr; registration number: KCT0001032; accessed on 18 March 2014). The study adhered to the Declaration of Helsinki and the Belmont Report.

A total of 100 participants aged 40–69 years, who visited the hospital for medical check-ups between August 2013 and November 2015, were enrolled after providing written informed consent. The participants were randomly selected from the community population. Individuals with diabetes, heart failure, myocardial infarction, or chronic kidney disease were excluded based on predefined exclusion criteria. Blood and spot urine samples were collected following an overnight fast of 8 h. Clinical laboratory parameters and serum 25(OH)D levels were measured using an enzymatic assay and a chemiluminescent immunoassay, respectively. In addition, clinical parameters related to liver function [alanine aminotransferase (ALT), aspartate aminotransferase (AST), gamma-glutamyl transpeptidase (γ-GTP)], kidney function [uric acid (UA)], cardiovascular health [high-sensitivity C-reactive protein (hs-CRP), hemoglobin A1c (HbA1c)], lipid metabolism [total cholesterol, high-density lipoprotein (HDL)-cholesterol, and triglycerides (TGs)] were also assessed. The concentrations of four HMs (Pb, Cd, Hg, and As) were quantified in blood and urine using inductively coupled plasma mass spectrometry.

### 2.2. Study-Group Classifications

The participants were classified based on serum 25(OH)D concentrations: 41 with SVDD (<10 ng/mL) and 59 with VDD (10–30 ng/mL). HM exposure was assessed by measuring the blood and urine concentrations of Pb, Cd, Hg, and As. Due to differing concentration units across the metals, the values were ranked to provide equal weighting. The sum of these ranks served as a composite exposure index [19]. Based on statistical significance, HM exposure was categorized into low (sum < 208) and high (sum > 208) exposure groups. Among the SVDD group, 18 participants had low HM exposure (SVDD–LHM), and within the VDD group, 28 had high HM exposure (VDD–HHM).

### 2.3. UHPLC-QTOF/MS Analysis of Plasma Samples

Plasma samples were stored at −80 °C and thawed at 4 °C before analysis. Quality control (QC) samples were prepared by mixing equal volumes of plasma samples. All analytical and QC samples were randomized during the preparation and analysis processes to minimize bias. Sodium formate solution (Sigma-Aldrich, St. Louis, MO, USA) was used for mass calibration, and a 100% methanol solvent blank (Merck, Darmstadt, Germany) assessed contamination. The lock mass, hexakis (2,2-difluoroethoxy) phosphazene (Apollo Scientific Ltd., Bredbury, UK), was co-ionized with analytes and used in place of an internal standard for accurate mass calibration.

Untargeted plasma metabolomic profiling was performed using ultra-high-performance liquid chromatography (UHPLC) coupled with quadrupole time-of-flight mass spectrometry (QTOF/MS). The system comprised a Thermo Scientific Dionex UltiMate 3000 UHPLC (Dionex Softron GmbH, Germany) with a Waters ACQUITY UPLC^®^ BEH C18 column (100 × 2.1 mm, 1.7 μm, 130 Å). Mobile phase A was water (J.T. Baker^®^, Avantor Performance Materials, LLC., Radnor, PA, USA) with 0.1% formic acid (Tokyo Chemical Industry Co., Tokyo, Japan), while mobile phase B was acetonitrile (Merck) with 0.1% formic acid. The injection volume and flow rate were 2 μL and 0.3 mL/min, respectively. Mass spectrometry was performed using a Bruker Compact™ QTOF (Bruker Daltonics GmbH & Co. KG, Bremen, Germany) with an electrospray ionization source in positive ion mode. A full scan was acquired across the 50–1000 *m*/*z* range. The MS/MS data were also collected in the same range, excluding 620–625 *m*/*z* to avoid fragmenting the lock mass at 622.0289 *m*/*z*.

### 2.4. Data Analysis

Raw UHPLC-QTOF/MS data underwent preprocessing prior to multivariate statistical analysis. Quantile normalization and log transformation were conducted using ProfileAnalysis version 2.1 (Bruker Daltonics GmbH & Co. KG, Bremen, Germany). Signal drift correction was performed using QC-based robust locally estimated scatterplot smoothing (LOESS) via the R package StatTarget (https://stattarget.github.io/docs, accessed on 20 September 2022). Pareto scaling was applied using SIMCA version 17.0 (Sartorius Stedim Data Analytics AB, Umeå, Sweden). The raw MS data, including both analytical and QC samples, were deposited in the Korea BioData Station (https://kbds.re.kr, accessed on 2 February 2023) under accession ID KAP240381.

Multivariate statistical analysis, including principal component analysis (PCA) and orthogonal partial least squares discriminant analysis (OPLS-DA), was conducted using SIMCA version 17.0. The OPLS-DA model quality was assessed by measuring R^2^X, (goodness of fit), R^2^Y (proportion of variance), and Q^2^ (predictive ability) [20]. The OPLS-DA model was validated using a permutation test with 100 iterations, while variable importance in projection (VIP) scores were obtained to assess metabolite contributions to class separation.

### 2.5. Putative Identification of Metabolites

Metabolic features were selected for putative identification based on a VIP score of ≥1.5, a mass tolerance of <5 ppm, and an mSigma value (isotopic pattern matching score) of <75. Metabolites were annotated at Level 2 according to the Metabolomics Standards Initiative [21]. MS/MS spectra were analyzed for metabolites using MetaboScape^®^ version 5.0 (Bruker Daltonics GmbH & Co. KG, Bremen, Germany). Database searches were conducted using MetaboScape’s internal library and external databases, including the Human Metabolome Database (HMDB, http://www.hmdb.ca, accessed on 8 July 2023), PubChem (http://pubchem.ncbi.nlm.nih.gov, accessed on 8 July 2023), ChEBI (https://www.ebi.ac.uk/chebi, accessed on 8 July 2023), and the Kyoto Encyclopedia of Genes and Genomes (KEGG, http://www.genome.jp/kegg, accessed on 8 July 2023).

### 2.6. Biomarker Analysis

To identify potential biomarkers, a biomarker analysis was conducted using MetaboAnalyst version 5.0 (https://www.metaboanalyst.ca, accessed on 10 March 2024). Univariate receiver operating characteristic (ROC) analysis was performed using a logistic regression algorithm to evaluate the effectiveness of metabolites as biomarkers. A 10-fold cross-validation was employed to train and evaluate the logistic regression model. Additionally, multivariate ROC analysis was performed using the partial least squares discriminant analysis (PLS-DA) algorithm. The area under the curve (AUC) was used to summarize model performance [22].

### 2.7. Statistical Analysis

All statistical analyses were performed using SPSS Statistics version 26.0 (IBM Corp., Armonk, NY, USA). The normality and homogeneity of variances were assessed using the Shapiro–Wilk and Levene’s tests, respectively. For continuous variables, one-way ANOVA or independent *t*-tests were used when assumptions were met. If not, the Kruskal–Wallis test, Mood’s median test, or Mann–Whitney *U* test was employed. Post hoc comparisons following ANOVA or Kruskal–Wallis were adjusted using the Bonferroni method. Categorical variables were analyzed using Pearson’s Chi-square or Fisher’s exact test. Correlations were assessed with Pearson’s or Spearman’s rank correlation coefficients. A *p*-value of <0.05 was considered statistically significant.

## 3. Results

### 3.1. Characteristics of the SVDD–LHM Group and the VDD–HHM Group

In total, 46 participants were divided into groups based on their HM exposure and 25(OH)D concentrations. The baseline data for the SVDD–LHM and VDD–HHM groups are presented in Table 1. Significant differences in HM exposures and 25(OH)D concentrations were observed between the two groups. No significant differences were found in the demographic or clinical variables, except for age. Age was higher in the VDD–HHM group compared to the SVDD–LHM group and showed a significant positive correlation with HM levels (Table S1).

### 3.2. Correlation Analysis Between 25(OH)D and HMs

**Figure 1:**
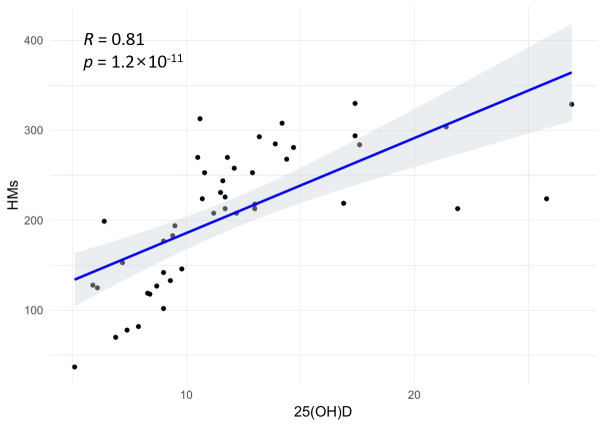
Spearman’s correlation analysis between the 25(OH)D concentration and HM exposures. 25(OH)D; 25-hydroxyvitamin D, HMs; heavy metals.

Correlation analysis examined the relationship between the 25(OH)D concentration and HM exposures (Figure 1), revealing a significant positive correlation (*R* = 0.81, *p* < 0.05). This suggests that, even under VDD conditions, elevated VD levels may facilitate the co-absorption of HMs. Accordingly, the participants were categorized into SVDD–LHM and VDD–HHM groups.

### 3.3. Untargeted Metabolic Analysis of Plasma Samples

**Figure 2:**
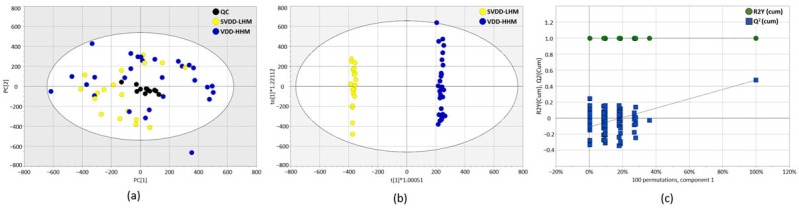
(**a**) Principal component analysis score plot derived from LC-MS/MS data of severe vitamin D deficiency–low level of heavy metals (SVDD–LHM) and vitamin D deficiency–high level of heavy metals (VDD–HHM). (**b**) The orthogonal partial least square-discriminant analysis (OPLS-DA) score plot. (**c**) A validation plot with 100 iterations for the OPLS-DA model. The y-intercepts of R^2^ and Q^2^ are 0.998 and -0.108, respectively. R^2^Y and Q^2^ are 0.998 and 0.477, respectively.

Untargeted metabolic analysis was conducted to investigate metabolite differences between the SVDD–LHM and VDD–HHM groups. The PCA score plot confirmed analytical precision and robustness by clustering the QC samples [23]. However, it did not exhibit a clear separation between the groups (Figure 2a). Consequently, OPLS-DA was applied to further explore metabolite differences. The OPLS-DA score plot revealed a distinct separation between the SVDD–LHM and VDD–HHM groups (R^2^X = 0.363, R^2^Y = 0.998, Q^2^ = 0.477) (Figure 2b). Model validity was confirmed by using permutation analysis (100 iterations), with y-intercept values for R^2^Y and Q^2^ of 0.998 and –0.108, respectively, indicating a valid model fit (Figure 2c).

### 3.4. Identification of Putative Metabolites

Significant metabolites were identified using the OPLS-DA model. Six putatively identified metabolites are listed in Table 2. Docosahexaenoic acid (DHA), phosphatidylcholine (PC) 20:5_18:3, PC 18:2_18:3, bilirubin, and eicosapentaenoic acid (EPA) were all higher in the VDD–HHM group compared to the SVDD–LHM group, whereas diacylglycerol (DAG) 15:0_18:3 exhibited the opposite trend. These metabolites are primarily involved in the biosynthesis of unsaturated fatty acids, glycerophospholipid metabolism, PC biosynthesis, and porphyrin metabolism.

### 3.5. Identification of Potential Biomarker

**Figure 3:**
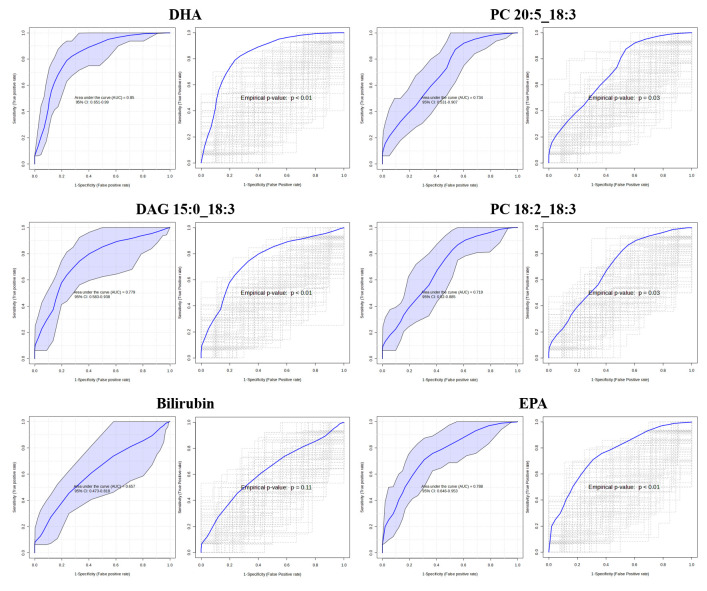
Receiver operating characteristic curve analysis of individual metabolites. The AUC (95% CI) and *p*-value for each metabolite are as follows: 0.850 (0.651–0.990) with *p* < 0.01 for DHA; 0.734 (0.531–0.907) with *p* = 0.03 for PC 20:5_18:3; 0.779 (0.583–0.938) with *p* < 0.01 for DAG 15:0_18:3; 0.719 (0.520–0.885) with *p* = 0.03 for PC 18:2_18:3; 0.657 (0.473–0.818) with *p* = 0.11 for bilirubin; and 0.788 (0.646–0.953) with *p* < 0.01 for EPA. AUC, area under the curve; CI, confidence interval; DHA, docosahexaenoic acid; PC, phosphatidylcholine; DAG, diacylglycerol; and EPA, eicosapentaenoic acid.

Biomarker analysis was conducted using ROC curves to compare the SVDD–LHM and VDD–HHM groups and to evaluate the metabolites as potential biomarkers. Six metabolites were analyzed through univariate ROC analysis using a logistic regression model (Figure 3). The AUC for DHA was 0.850, while the AUC values for PC 20:5_18:3, DAG 15:0_18:3, PC 18:2_18:3, and EPA were 0.734, 0.779, 0.719, and 0.788, respectively, and all were statistically significant. The bilirubin values were not significant. The sensitivity and specificity values and the 95% confidence intervals (CIs) for these six metabolites are presented in Table S2.

Multivariate ROC analysis was also conducted to evaluate the AUC for various metabolite combinations. Bilirubin, which was not significant in the univariate ROC analysis, was excluded. The model comprising a combination of these five metabolites achieved the highest AUC of 0.817 (95% CI: 0.651–0.974) (Figure S1a), with a predictive accuracy of 76.0% (Figure S1b). The AUC of the ROC model generated using only DHA was 0.850, suggesting that DHA may be associated with predicting VD activity in response to high HM exposure.

## 4. Discussion

This study examined the relationship between HM and VD levels from a metabolic perspective, as well as the association between VD-induced antioxidant activity and this relationship. Our findings suggest that increased VD levels may lead to elevated HM accumulation, which in turn promotes ROS production. This ROS production may subsequently activate the antioxidant defense system associated with VD. A positive correlation was also observed between VD levels and HM exposures, suggesting that VD may enhance HM absorption. Consequently, six metabolites (DHA, PC 20:5_18:3, DAG 15:0_18:3, PC 18:2_18:3, bilirubin, and EPA) were putatively identified and found to be associated with the VD-related antioxidant pathway.

In this study, all of the participants were recruited from the general population of a local community, and their serum 25(OH)D concentrations were below 30 ng/mL. This is a primary limitation of the study. However, this finding aligns with the national average 25(OH)D concentration of 17.1 ± 0.1 ng/mL reported in the Korea National Health and Nutrition Examination Survey, which included participants from all 16 provinces [24]. As sunlight exposure is the main source of VD synthesis, factors such as seasonal variation, occupational setting, and socioeconomic status may act as confounders. Although 25(OH)D concentrations tend to rise during summer, the national average among Koreans remains low at 20.6 ± 0.2 ng/mL in that season [25]. Furthermore, stratification by occupation (indoor vs. outdoor), walking frequency, education level, and income showed that most subgroups still had mean 25(OH)D concentrations below 20 ng/mL. Even in outdoor workers, the average was 21.6 ± 0.4 ng/mL [24], which is classified as VDD under this study’s criteria. Consequently, even when accounting for various factors that influence VD levels in the human body, the majority of Koreans are classified within the VDD group, which suggests that the VD levels of our study subjects are representative of the general Korean population.

Blood Pb, Cd, and Hg concentrations, along with urinary As, were measured to assess HM exposure. Blood Pb levels, which reflect bone Pb concentrations due to bone storage of Pb, are commonly used for exposure monitoring [26,27]. Since there is no significant difference in blood and urine Cd concentrations in the general population, blood Cd levels were analyzed [28]. Blood Hg levels were also measured because dietary intake is the primary exposure route [29]. Urinary As concentrations were creatinine-adjusted due to As’s short blood half-life of approximately 1 h [30].

Demographic and clinical variables associated with endogenous VD and HM concentrations were selected based on their known relevance to the VD status and exposure to HMs [31,32,33,34]. All variables were controlled in both groups. However, age was higher in the high-HM group, and a significant positive correlation was observed between age and HM exposure (Table S1), consistent with previous studies attributing increased HM levels in older adults to bioaccumulation. [35,36]. In addition, both VDD and HM exposure have been linked to hypertension development [31,32]. In our study, individuals with hypertension were present in both the SVDD–LHM and VDD–HHM groups, but the prevalence did not differ significantly between groups. Although hypertension may influence metabolic responses, neither 25(OH)D levels nor HM concentrations were significantly associated with hypertension in our study population (*R* = 0.0708, *p* = 0.64 for 25(OH)D; *R* = 0.0149, *p* = 0.92 for HM), indicating that hypertension likely did not serve as a confounding factor.

**Figure 4:**
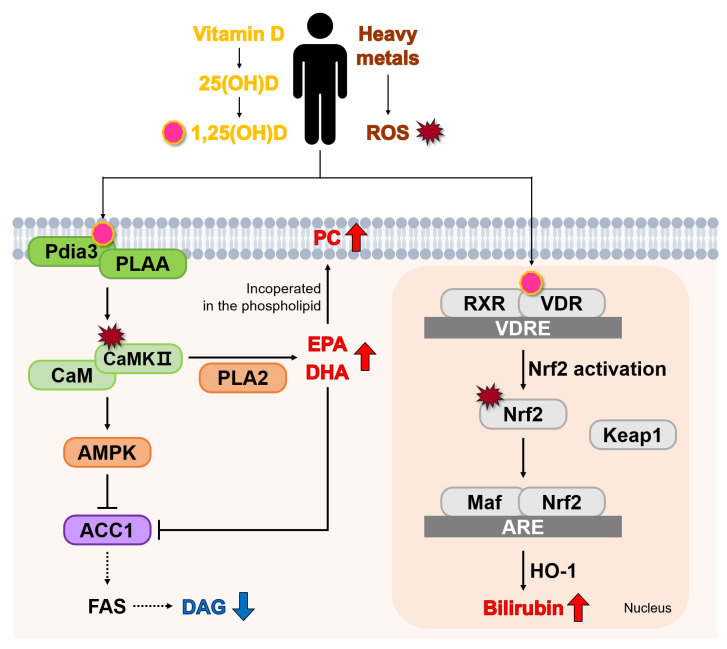
Changes in metabolites induced by the antioxidant activity of vitamin D in response to heavy metals. The arrows indicating increases or decreases in metabolites represent the directional trends in the VDD–HHM group relative to the SVDD–LHM group. 1,25(OH)D, 1,25-dihydroxy vitamin D; ROS, reactive oxygen species; Pdia3, protein disulfide isomerase family A member 3; PLAA, phospholipase A2-activating protein; CaM, Ca^2+^/calmodulin; CaMKII, CaM-dependent protein kinase II; PLA2, phospholipase A2; AMPK, AMP-activated protein kinase; ACC1, acetyl-CoA carboxylase1; TCA cycle, tricarboxylic acid cycle; acetyl-CoA, acetyl coenzyme A; malonyl-CoA, malonyl coenzyme A; RXR, retinoid X receptor; VDR, vitamin D receptor; VDRE, vitamin D response element; Maf, musculoaponeurotic fibrosarcoma; Nrf2, nuclear factor erythroid 2-related factor 2; Keap1, kelch-like ECH-associated protein 1; ARE, antioxidant response element; and HO-1, heme oxygenase-1.

Six metabolites were putatively identified as exhibiting differences between the SVDD–LHM and VDD–HHM groups. Lipid-related metabolites (DHA, PC 20:5_18:3, DAG 15:0_18:3, PC 18:2_18:3, and EPA) can vary based on the different cholesterol and lipid types present in the body, including total cholesterol, HDL-cholesterol, and TGs. However, no significant differences in total cholesterol, HDL-cholesterol, or TGs were observed between the groups. Bilirubin, a marker that may rise due to liver injury, was also assessed. Liver function markers (ALT, AST, and γ-GTP) also did not differ significantly between the groups, indicating that the observed metabolite changes were not due to clinical laboratory findings. Therefore, only the effects of VD and HM were considered. We examined metabolic alterations through the VD activation pathway influenced by HM exposure and investigated metabolite interrelationships based on the six putatively identified metabolites (Figure 4).

VD is metabolized in the liver to form 25(OH)D, which is subsequently converted to 1,25-dihydroxyvitamin D (1,25(OH)D) by the kidneys [37]. The active form, 1,25(OH)D, selectively interacts with the vitamin D receptor (VDR) and the membrane-associated receptor Pdia3 (protein disulfide isomerase family A member 3) [38]. Furthermore, 1,25(OH)D binding to Pdia3 stimulates the phospholipase A2 activating protein (PLAA) and Pdia3 interaction, activating Ca^2+^/calmodulin-dependent protein kinase II (CaMKII), which mediates signaling from PLAA to phospholipase A2 (PLA2) [38,39]. CaMKII activation increases in pro-oxidant environments [40], and HM-induced ROS contribute to this process [41]. PLA2 can produce EPA and DHA by hydrolyzing the acyl bond in the phospholipid membrane at sn-2 [42]. EPA and DHA are essential omega-3 polyunsaturated fatty acids (PUFAs) that elicit antioxidative and anti-inflammatory effects [43] and may be particularly effective in mitigating oxidative stress.

CaMKII—activated by 1,25(OH)D—and ROS, induced by HMs, regulate lipid metabolism by promoting EPA and DHA production and by phosphorylating AMP-activated protein kinase (AMPK) [44,45]. AMPK activation inhibits fatty acid (FA) synthesis via the phosphorylation of acetyl-CoA carboxylase 1 (ACC1), an enzyme that catalyzes the conversion of acetyl-CoA to malonyl-CoA—a key step in de novo FA synthesis [46,47]. Consequently, if HM-induced ROS further enhances the VD-related antioxidant pathway, fatty acid synthase (FAS) activity may be inhibited, reducing the availability of fatty acyl-CoA and fatty acids for lipid synthesis, and thereby decreasing diacylglycerol (DAG) production. Lipid changes were indeed observed in metabolic profiling and varied based on 25(OH)D concentrations and HM exposure. Jeromson et al. [48] reported that EPA and DHA increased the ratio of lipid species containing long chains and double bonds. Jeromson et al. (2018) also noted that long-chain PUFAs are incorporated into phospholipid species such as PC and phosphatidylethanolamine. These findings are similar to our results, as elevated long-chain PC (PC 20:5_18:3 and PC 18:2_18:3) levels were observed in the VDD–HHM group with high EPA and DHA levels. PUFAs, particularly DHA, enhance biological membrane stability by remodeling the plasma membrane lipidome [49]. These findings suggest that PUFAs help maintain membrane integrity by integrating into phospholipids, protecting cells from HM-induced membrane damage.

When 1,25(OH)D binds to the VDR, it forms a heterodimer with the retinoid X receptor. This complex interacts with vitamin D response elements (VDREs) in the promoter regions of VD-regulated genes, activating gene targets or initiating intracellular signaling pathways [50]. VD also mitigates oxidative stress by upregulating the nuclear factor erythroid 2-related factor 2 (*Nrf2*) pathway [1,51]. *Nrf2* is an antioxidant gene regulated by kelch-like ECH-associated protein 1 (Keap1) and is activated to reduce ROS [52]. It binds to the antioxidant response element, promoting the expression of heme oxygenase-1 and the production of bilirubin, a known endogenous antioxidant. This pathway can also be activated by HM-induced ROS generation [53]. Therefore, elevated bilirubin levels under higher HM exposure may reflect enhanced antioxidant gene expression and support a homeostatic antioxidant response under oxidative stress conditions.

Univariate ROC analysis was conducted to evaluate the predictive performance of six metabolites related to VD-mediated antioxidant activity under HM exposure. Five metabolites (DHA, PC 20:5_18:3, DAG 15:0_18:3, PC 18:2_18:3, and EPA) showed an AUC greater than 0.7. Multivariate ROC analysis was also performed using combinations of metabolites to improve predictive accuracy (Figure S1). The results show that the model incorporating all five metabolites yielded the highest AUC (0.817; 95% CI, 0.651–0.974). However, DHA alone had an even higher AUC (0.850; 95% CI, 0.651–0.990), indicating that it may be the most robust single predictor of antioxidant activity induced by VD in response to HM exposure. In contrast, bilirubin did not show a statistically significant result in ROC analysis (AUC = 0.657, *p* = 0.11), possibly due to its high inter-individual variability. Notably, the standard deviation of bilirubin was more than twice that of the other five metabolites in each group, suggesting that this high dispersion may have impaired its predictive performance.

Unfortunately, dietary intake data were not collected in this study, limiting our ability to account for confounding factors such as fish consumption or dietary supplements that could influence EPA and DHA levels. Nevertheless, a strong positive correlation was observed between VD concentrations and HM levels in the body. To the best of our knowledge, this is the first metabolomics-based study to investigate the VD-mediated antioxidant activation pathway in response to HM exposure under conditions of VDD, underscoring the novelty and importance of our findings.

Notably, the comparison between the SVDD–LHM and VDD–HHM groups revealed distinct metabolomic responses, suggesting that VD may enhance antioxidant activity when exposed to HMs. This provides a novel insight into the physiological role of VD. Moreover, our findings offer metabolomics-based evidence complementing previous epidemiological and animal studies, which reported concurrent increases in VD levels and HMs. The identified metabolites may serve as potential biomarkers indicative of the body’s defense mechanisms in response to HM exposure under varying VD statuses. These findings suggest that HM exposure levels should be considered when evaluating interventions targeting the antioxidant effects of VD in clinical and public-health settings. External validation using a larger cohort with adequate VD levels is necessary to improve statistical power for clinical applications.

## 5. Conclusions

This study was based on the hypothesis that increased VD concentrations promote HM uptake, thereby activating antioxidant pathways associated with VD. We putatively identified six endogenous human metabolites (DHA, PC 20:5_18:3, DAG 15:0_18:3, PC 18:2_18:3, bilirubin, and EPA) which were associated with metabolic pathways that were involved in enhancing antioxidant responses to HM exposure. Among these, DHA exhibited a ROC-AUC greater than 0.85, suggesting its potential as a biomarker for antioxidant activity under conditions of varying VD status and HM exposure.

These findings offer novel insight into the activation of antioxidant pathways through the interaction between VD and HM and suggest a metabolomics-based approach to evaluating VD’s antioxidant function. For the clinical application of these findings, further studies are needed, with more stringent control of confounding variables and the inclusion of a cohort with adequate VD levels.

## Figures and Tables

**Table 1:** Baseline information for the SVDD–LHM and VDD–HHM groups.

Variables	SVDD–LHM	VDD–HHM	*p*-Value *
*n*	18	28	
25(OH)D, ng/mL	8.4 [5.110–9.8]	13.0 [10.5–26.9]	0.000
Heavy metals	127.5 [37.0–199.0]	255.50 [208.0–330.0]	0.000
Age, year	42.0 [32.0–60.0]	53.0 [32.0–65.0]	0.001
Male, *n*	6	11	0.761
Smoking, *n*	1	6	0.417
Drinking, *n*	11	13	0.378
Hypertension, *n*	5	7	0.834
BMI	24.1 [17.9–37.4]	25.4 [21.5–32.0]	0.862
ALT	20.0 [12.0–103.0]	20.5 [12.0–128.0]	0.397
AST	23.0 [14.0–69.0]	25.5 [16.0–105.0]	0.295
γ-GTP	19.0 [9.0–105.0]	24.5 [9.0–129.0]	0.710
Uric acid	4.6 [2.3–7.4]	5.1 [2.1–7.5]	0.178
hs-CRP	0.6 [0.2–2.6]	0.4 [0.1–4.3]	0.362
HbA1c	5.4 [5.0–5.7]	5.5 [4.8–6.1]	0.260
Total cholesterol	184.5 [134.0–313.0]	195.0 [151.0–236.0]	0.831
HDL-cholesterol	57.5 [34.0–85.0]	53.5 [29.0–94.0]	0.860
TGs	96.0 [34.0–536.0]	108.5 [3.9–348.0]	0.770

These data are presented as median [range]. SVDD, severe vitamin D deficiency; LHM, low level of heavy metals; VDD, vitamin D deficiency; HHM, high level of heavy metals; 25(OH)D, 25-hydroxyvitamin D; BMI, body mass index; ALT, alanine aminotransferase; AST, aspartate aminotransferase; GTP, guanosine triphosphate; hs-CRP, high-sensitivity C-reactive protein; HbA1c, hemoglobin A1c; HDL, high-density lipoprotein; TGs, triglycerides. * A *p*-value of <0.05 was considered statistically significant. The *p*-value was calculated using the independent *t*-test or Mann–Whitney *U* test for continuous variables, and Pearson’s chi-squared test or Fisher’s exact test were used for categorical variables.

**Table 2:** A list of significantly altered human endogenous metabolites in the VDD–HHM group compared to the SVDD–LHM group.

VIP Score	RT	*m*/*z*	Name	HMDB ID	Related Pathway	*p*-Value *	Trend
2.47	10.27	329.2478	Docosahexaenoic acid	HMDB0002183	Biosynthesis of unsaturated fatty acids	0.000	↑
2.29	16.62	802.5380	PC 20:5_18:3	HMDB0008501	Glycerophospholipid metabolism	0.002	↑
2.25	13.02	577.4838	DAG 15:0_18:3	HMDB0007075	Phosphatidylcholine biosynthesis	0.002	↓
2.02	16.13	780.5552	PC 18:2_18:3	HMDB0008624	Glycerophospholipid metabolism	0.004	↑
1.91	6.01	585.2719	Bilirubin	HMDB0000054	Porphyrin metabolism	0.049	↑
1.55	9.83	303.2309	Eicosapentaenoic acid	HMDB0001999	Biosynthesis of unsaturated fatty acids	0.001	↑

VIP, variable importance in the projection; RT, retention time; *m*/*z*, mass-to-charge ratio; HMDB, Human Metabolome Database; PC, phosphatidylcholine; DAG, diacylglycerol. * A *p*-value of <0.05 was considered statistically significant and was calculated using either the independent *t*-test or the Mann–Whitney *U* test.

## Data Availability

The original data presented in the study are openly available in the Korea BioData Station (https://kbds.re.kr, accessed on 2 February 2023) at accession ID KAP240381.

## Supplementary Materials

The following supporting information can be downloaded at: https://www.mdpi.com/article/10.3390/metabo15070440/s1, Figure S1: Multivariate receiver operating characteristic curve analysis for five metabolites; Table S1: Correlation analysis between age and clinical variables in all participants; and Table S2: Logistic regression model data for six metabolites.
