# Supplementary material for: Impact of Heavy Metals on the Antioxidant Activity of Vitamin D: A Metabolic Perspective
Source: Metabolites. 2025 Jul 1;15(7):440. doi: 10.3390/metabo15070440 (PMC12300271; doi:10.3390/metabo15070440)
Supplement: Supplementary file 1 [file metabolites-15-00440-s001.zip › metabolites-3705886_Supplementary File.pdf]

Table S1. Correlation analysis between age and clinical variables in all participants.

Variables	Age	
	R[a]	*p*-value[*]
VD	0.326	0.027
HMs	0.519	0.001
ALT	-0.066	0.664
AST	-0.019	0.903
γ-GTP	-0.185	0.220
Total cholesterol	0.215	0.152
HDL-cholesterol	0.151	0.316
TGs	-0.192	0.201
Uric acid	-0.126	0.406
hs-CRP	0.078	0.604
HbA1c	0.245	0.101
WBC	0.083	0.589
RBC	0.105	0.493
Hemoglobin	0.065	0.673
Hematocrit	0.030	0.843
MCV	-0.146	0.338
MCH	-0.063	0.679
MCHC	0.144	0.346
Platelet	-0.116	0.450

VD, vitamin D; HMs, heavy metals; ALT, alanine aminotransferase; AST, aspartate aminotransferase; GTP, guanosine triphosphate; TGs, triglycerides; HDL, high-density lipoprotein, hs-CRP, high-sensitivity C-reactive protein; HbA1c, hemoglobin A1c; WBC, white blood cells; RBC, red blood cells; MCV, mean corpuscular volume; MCH, mean corpuscular hemoglobin; MCHC, mean corpuscular hemoglobin concentration.

[a]The correlation coefficients were calculated using Pearson's or Spearman's rank correlation analysis.

Table S2. Logistic regression model data for six metabolites.

Metabolites	Sensitivity (95% CI)		Specificity (95% CI)	
	Discovery	Validation	Discovery	Validation
Docosahexaenoic acid	0.817 (0.770–0.865)	0.821 (0.821–0.963)	0.772 (0.707–0.836)	0.778 (0.586–0.970)
PC 20:5_18:3	0.937 (0.906–0.967)	0.964 (0.964–1.000)	0.475 (0.398–0.552)	0.500 (0.269–0.731)
DAG 15:0_18:3	0.639 (0.580–0.698)	0.679 (0.679–0.852)	0.747 (0.680–0.814)	0.778 (0.586–0.970)
PC 18:2_18:3	0.730 (0.675–0.785)	0.750 (0.750–0.910)	0.593 (0.517–0.668)	0.611 (0.386–0.836)
Bilirubin	0.623 (0.563–0.683)	0.643 (0.643–0.820)	0.599 (0.523–0.674)	0.611 (0.386–0.836)
Eicosapentaenoic acid	0.710 (0.654–0.766)	0.714 (0.714–0.882)	0.771 (0.707–0.836)	0.778 (0.586–0.970)

CI, confidence interval; PC, phosphatidylcholine; DAG, diacylglycerol.

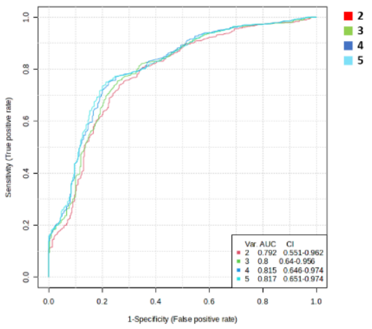

(**a**)

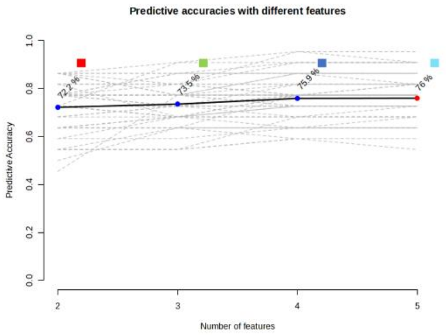

(**b**)

Figure. S1 Multivariate receiver operating characteristic curve analysis for five metabolites. (a) A model combining metabolites generated using the PLS-DA method. (b) Predictive accuracy for the combination of metabolites. The labels indicate the number of metabolites used to create the AUC, as represented by the colors. Var., variables; AUC, area under the curve; CI, confidence interval
